# Ultrasound Assessment of Honey Using Fast Fourier Transform

**DOI:** 10.3390/s21206748

**Published:** 2021-10-11

**Authors:** Montaña Rufo, Antonio Jiménez, Jesús M. Paniagua, Alberto González-Mohíno

**Affiliations:** 1 Department of Applied Physics, School of Technology, University of Extremadura, Avenida de la Universidad s/n, 10003 Cáceres, Spain; mmrufo@unex.es (M.R.); paniagua@unex.es (J.M.P.); 2 Research Institute of Meat and Meat Product, University of Extremadura, Avenida de la Universidad s/n, 10003 Cáceres, Spain; albertogj@unex.es; 3 Department of Food Technology, Faculty of Veterinary, University of Extremadura, Avenida de la Universidad s/n, 10003 Cáceres, Spain

**Keywords:** non-destructive ultrasound, honey, fast Fourier transform (FFT), texture profile analysis (TPA)

## Abstract

Ultrasound inspection permits the characteristics of some foodstuffs to be determined easily and cheaply. This experimental study included the determination of various ultrasound parameters provided by the fast Fourier transform (FFT) which had not previously been considered in testing the physical properties of different varieties of honey. These parameters are practically independent of the criteria adopted for their calculation, unlike other ultrasound variables such as pulse velocity or attenuation whose determination can vary depending on those criteria. The study was carried out on four varieties of honey (Eucalyptus, Heather, Thyme, and Thousand Flowers) using 500-kHz transducers. A simultaneously performed honey texture analysis (Texture Profile Analysis-TPA) showed significant linear correlations between the ultrasound variables provided by FFT and the texture parameters. The FFT parameters distinguished between each of the four honey varieties studied.

## 1. Introduction

Honey is a food made by the bee *Apis mellifera* transforming the nectar of the flowers and specific substances of their own. It is traditionally used as a sweetener and for therapeutic applications. Honey is a very complex product whose nutritional composition is mainly of monosaccharides which include a mixture of glucose and fructose, besides water and other minor components such as proteins, enzymes, amino acids, phenolic compounds, minerals, vitamins, and organic acids [1]. Interest in different types of honey has increased due to their different nutritional, sensorial and possible therapeutic characteristics [2]. Of the two varieties of honey—multifloral and monofloral—the latter is more valued by consumers [3].

The flavor, aroma, and phenolics content of honeys is strongly associated with their pollen, nectar, resin, oil, botanical source, geographical area, environmental storage conditions, and the bee subspecies involved in their production. Consequently, honeys with different floral origins present distinct bioactive properties [4], so that an ability to differentiate between varieties of honey, characterizing them and determining their quality, would be of acute importance for consumers. In recent years, various efforts have been made to address the authenticity [5], traceability [6], and intrinsic quality of honeys by applying techniques such as chromatography, nuclear magnetic resonance (NMR), and isotope-ratio mass spectrometry (IRMS) [7]. Most of these techniques, however, are destructive, and others, such as microscopic analysis, are not valid for all types of honey varieties [8].

Several new non-destructive techniques (X-ray, NIR spectroscopy, ultrasound, etc.) have been adapted for the measurement of a wide range of quality parameters during food processing [9]. Examples of ultrasound inspection in particular can be found in various studies in the recent literature—monitoring the pork loin cooking process [10], characterizing edible oils [11], and monitoring coagulation in cheesemaking [12,13]. Some studies have used ultrasound parameters to characterize honey. For instance, Singh et al. [14] were able to prove adulterations of honey using the speed of ultrasound passing through honey samples. These kinds of parameters have also been used recently to distinguish honeys at different temperatures [15,16]. González-Mohino et al. [17] used ultrasound attenuation to characterize different varieties of honey.

The objective of the present study was to use different ultrasound parameters extracted by the fast Fourier transform (FFT) technique to characterize and distinguish different types of honey (Eucalyptus, Heather, Thyme, and Thousand Flowers). This transform gives a frequency domain representation of the amplitude and phase of a continuous signal acquired in the time domain, speeding up the process in comparison with the calculation of discrete Fourier transform (DFT) which would be computationally very intensive. Thus, the procedure used to calculate and plot the FFT identifies the frequency range covering the received ultrasound pulse signal, particularly the 25th, 50th, and 75th percentile frequencies corresponding to the cumulative frequency periodogram and the center frequency (that of maximum amplitude) for each sample. In addition, these parameters were correlated with the data of a textural study that had been carried out in samples of these kinds of honey [17]. To the best of our knowledge, there have been no previous studies in the scientific literature that correlate data from these two techniques. The study is thus intended to contribute to knowledge about the use of the FFT in honey characterization, obtaining parameters that have not previously been considered for this kind of product. In addition, the determination of the aforementioned FFT parameters is practically independent of the criteria adopted by the analyst for their calculation. This is unlike the case with other ultrasound parameters such as velocity or attenuation whose determination can vary depending on the criteria adopted [18]. We believe that the information obtained in this work is a first step towards implementing effective and rapid methods that can be used to verify the authenticity of a product as highly valued as honey. It provides objective tools to control the quality of different varieties of honey, based on the determination of non-destructive parameters in them. The existence of mathematical relationships between some of the studied non-destructive parameters and other destructive ones, indicators of the quality of honey, will specify the benefits that the new techniques should offer so that they can totally or partially replace conventional ones. We must add that ultrasounds do not physically, chemically, or biologically alter the product. In this way, the thematic scope of this paper has a vast field of applications, is innovative and, of course, has a great potential impact, thus contributing to the food safety and quality needs.

## 2. Materials and Methods

Four varieties of honey were analyzed—three of them monofloral, Eucalyptus, heather, and, Thyme and one multifloral, Thousand Flowers. They all belong to a brand “Sabores del Guijo” with a Designation of Origin and are produced in Guijo de Santa Bárbara, Cáceres (Spain). The honey was heated up to 45 °C and ultrasonic inspection was carried out at different temperatures, 45 °C, 40 °C, 35 °C, 30 °C, and 25 °C while the samples were cooled. Two samples of every varieties of honey were analyzed.

**Figure 1:**
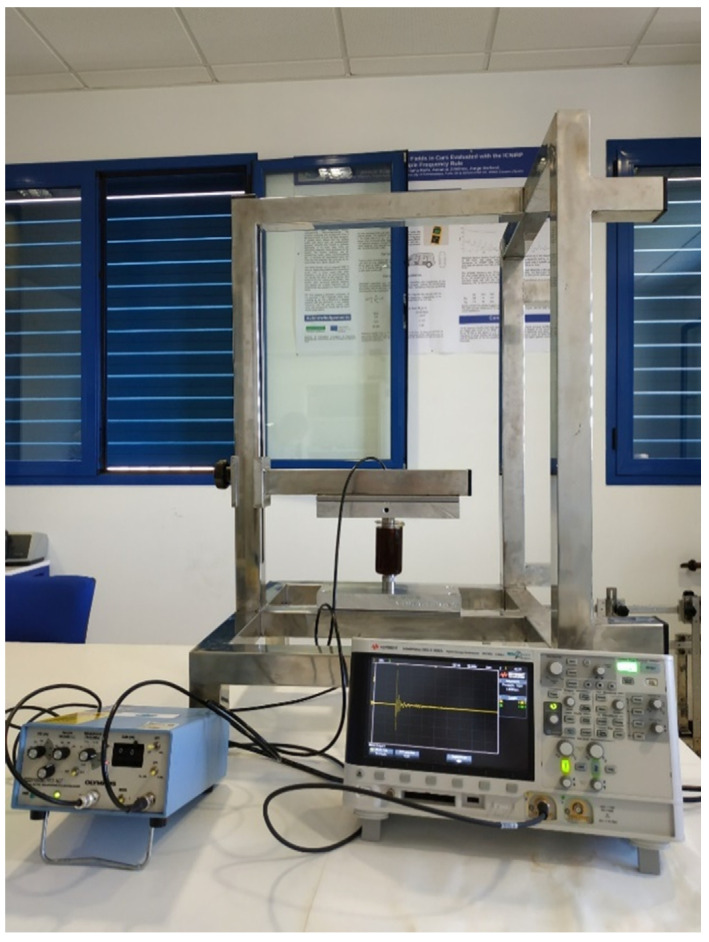
Photograph of the measurement set-up for the determination of ultrasound parameters.

To determine the ultrasound parameters, two Olympus Panametrics-NDT Model V318-SU (Olympus NDT Inc, Waltham, MA*,* USA), piezoelectric transducers (frequency 500 kHz, 19 mm in diameter, 61.93% at 6 dB bandwidth) were used to transmit the signals in through-transmission (T-T) mode. It is known that this and the pulse-echo mode yield very similar results [13], so that one can say that using one or the other is of little relevance. Figure 1 shows the experimental set-up. At the back of the apparatus, the metal structure designed to arrange the two transducers in such a way as to ensure their perfect face-to-face alignment can be seen, with the sample placed in the middle. In this case, the receiver transducer was placed in the upper part of the jar slightly submerged in the honey sample, and the emitter was always placed against the bottom glass of the jar. Near-field length (*N* = 2.12 cm) and beam spread angle (*φ* = 15.84°) have also been studied considering an ultrasound pulse velocity of 2125 m/s. The transducers were connected to an Olympus Model 5077PR (Olympus NDT Inc, Waltham, MA*,* USA) Pulser–Receiver shown at the left of Figure 1, which emitted and received the signal from the transducers. On this signal’s reception, the InfiniiVision DSO-X 3032A oscilloscope (Keysight Technologies Malaysia Sdn Bhd, Penang, Malaysia) shown was responsible for its acquisition and digitalization. More methodological details can be consulted in González-Mohino et al. [17]. The separation between transducers was 8.5 cm, ensuring measurements in the far field.

### 2.1. Ultrasound Parameters

Ultrasound parameters were determined from the fast Fourier transform (FFT) of the A-scan. In particular, these parameters were the central frequency of the FFT, and its 25th, 50th, and 75th percentile frequencies.

**Figure 2:**
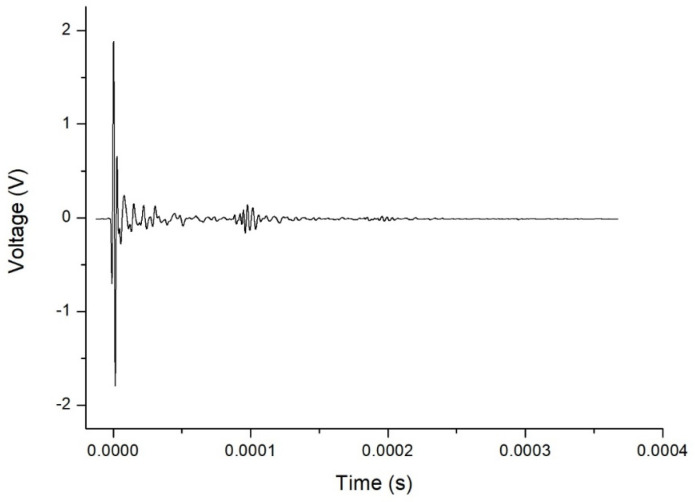
Typical A-scan (amplitude vs. time) obtained for Eucalyptus honey.

**Figure 3:**
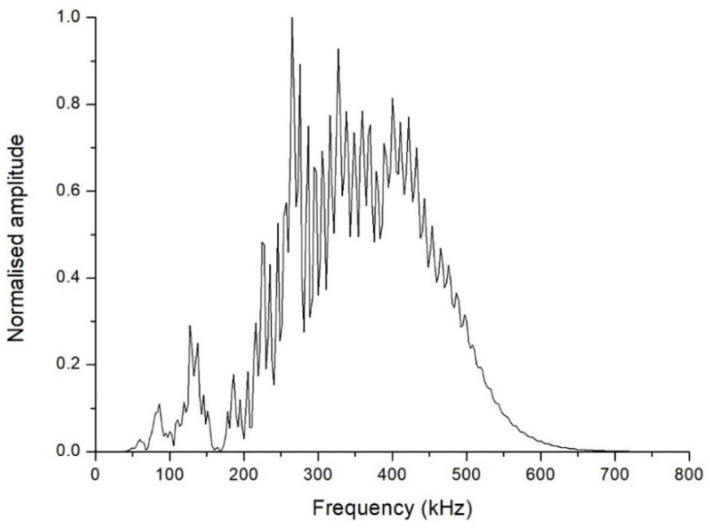
Fast Fourier transform of the signal shown in Figure 2.

By way of example, Figure 2 shows an A-scan obtained from the ultrasound testing of a Eucalyptus honey sample, in which consecutive echoes caused by reflections between the transducers can be seen. The FFT technique used to calculate the aforementioned parameters is an algorithm that speeds up the process of determining the amplitude and phase in the frequency domain of a continuous signal acquired in the time domain. The FFT obtained from the aforementioned A-scan is shown in Figure 3. The plot of the amplitude of the signal in the frequency domain allows one to visualize the frequency range of the received signal, and the different contributions of each frequency to the signal obtained after it has passed through the sample. In this representation, the central frequency is that with maximum amplitude. From this spectrum, one can derive certain properties of the sample under study.

**Figure 4:**
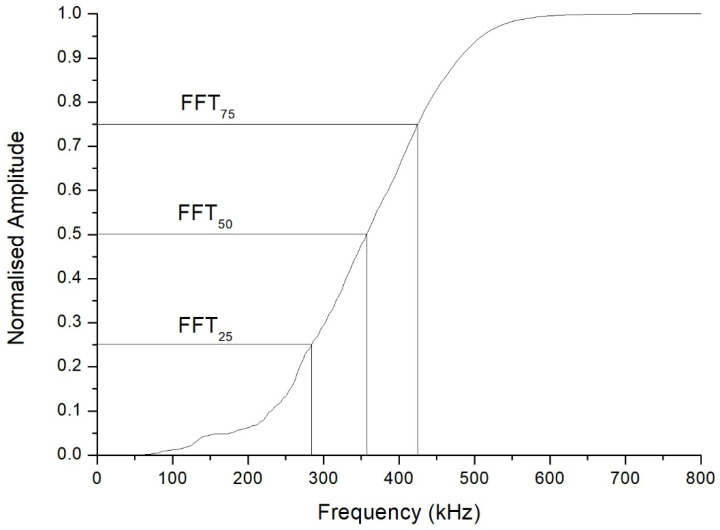
Cumulative frequency periodogram of the fast Fourier transform shown in Figure 3. The 25th, 50th, and 75th percentiles of the frequencies are indicated.

One sees in Figure 3 that the central frequency is approximately 265 kHz, a frequency clearly lower than the 500 kHz central frequency of the transducers. This fact was the case in all the inspections carried out. Furthermore, unlike what might have been expected a priori, the FFT plot is far from approaching a Gaussian shape. This is why it was considered more useful to compute the cumulative periodogram for each sample, and take as references the values corresponding to the 25th, 50th, and 75th percentiles of the received signal. The interpretation is that, if the 25th percentile of the cumulative frequency is at x kHz for a particular inspection, this means that 25% of the received signals had frequencies below x kHz. As an example, Figure 4 shows the cumulative frequency periodogram corresponding to the FFT of Figure 3. For each A-scan and subsequent FFT and frequency periodogram, the values of the different percentiles of the signals received are denoted as FFT_25_, FFT_50_, and FFT_75_. By way of example, the values of these percentiles of the signals received and shown in Figure 4 were 286 kHz for FFT_25_, 359 kHz for FFT_50_, and 427 kHz for FFT_75_.

### 2.2. Texture Analysis

The texture analysis was carried out using the procedure described in a previous paper [17]. The parameters measured were: adhesiveness (Nxs), chewiness (N), hardness, and gumminess (N). The study was carried out in triplicate at three temperatures; 25 °C, 30 °C, and 35 °C, which are usually the storage temperatures of honey [17].

## 3. Results and Discussion

### 3.1. Fast Fourier Transform

**Figure 5:**
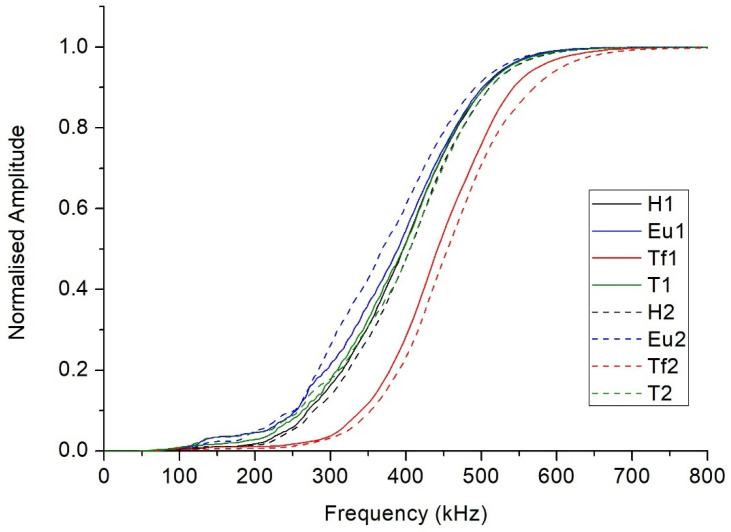
The cumulative frequency periodograms corresponding to the honey samples at 40 °C. (H = Heather, Eu = Eucalyptus, Tf = Thousand Flower, and T = Thyme).

Figure 5 shows the cumulative frequency periodograms corresponding to the honey samples at 40 °C. As can be observed, the samples presented quite different behaviors, with that of a Thousand Flowers being particularly distinguishable. In overall terms, the Thousand Flowers variety favors the transmission of higher frequency ultrasound waves than those allowed by the other varieties. For example, 70% of the Thousand Flowers variety’s frequency components are below 500 kHz, while this proportion is clearly greater in the other three varieties.

Table 1 lists the results for the FFT central frequency and the 25th, 50th, and 75th percentiles for each variety and batch of honey. As can be seen, the central frequency is lower than the nominal frequency of the transducers for all the samples. Indeed, FFT_50_ is lower than 500 kHz in most cases. Unfortunately, we could find no published data with which to contrast the present results. One observes in the data of the table that, at temperatures of 35 °C and above, the separation of the Thousand Flowers honey frequency values from those of the other varieties is clear, while these other varieties (Eucalyptus, Heather, and Thyme) differ very little from each other.

**Figure 6:**
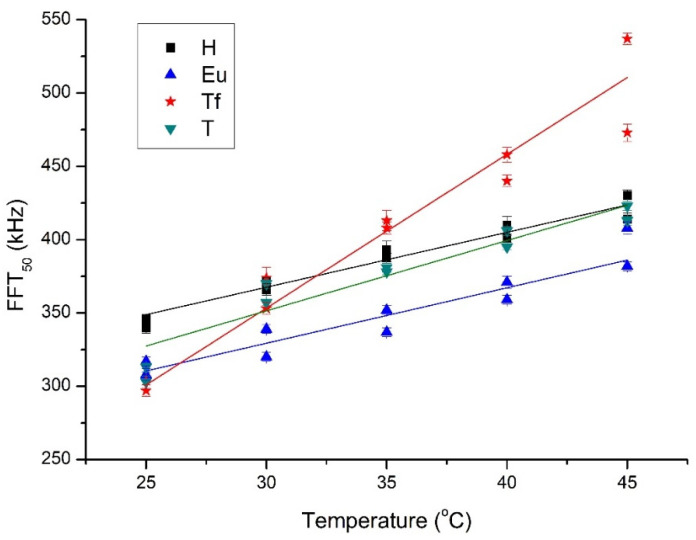
Evolution of FFT_50_ with temperature for both batches and every type of honey (H = Heather, Eu = Eucalyptus, Tf = Thousand Flower, T = Thyme).

Figure 6 shows the evolution of the FFT_50_ during the aforementioned cooling process for both batches of the four varieties of honey. The trend of higher ultrasound frequencies is more poorly transmitted as the honey cools can be clearly seen. While this trend is true for all four varieties studied, there are differences depending on the variety. This pattern for FFT_50_ is repeated for FFT_25_ and FFT_75_, and may be related to less presence of solids (crystals) in the samples at the warmer temperatures, thus allowing higher frequencies to pass through more easily [19]. Table 2 lists the linear correlations parameters (slope, m; intercept, n; correlation coefficient, r) between the FFT_25_, FFT_50_, FFT_75_, FFTcentral, and the temperature of the honey. As can be seen in the table, all of these correlations are strong. As was to be expected due to the aforementioned non-Gaussian behavior, the correlations related to FFTcentral are weaker than those of the rest of the FFT parameters. The correlation parameters allow one to distinguish between different types of honey. In particular, the slope, m, distinguishes Thousand Flowers from the other three honeys for every FFT percentile, e.g., for FFT_25_, m is 10 for Thousand Flowers but around 4 for the others (Thyme, Eucalyptus, and Heather). A close examination of the intercept, n, lets one discriminate among the other three honeys. In particular, the intercept, n, for FFT_25_, FFT_50_ and FFT_75_ is able to distinguish Heather from Thyme, being greater for the former in all three cases. Its FFT_50_ and FFT_75_ values allow Eucalyptus and Heather to be differentiated, with it again being greater for Heather. Hence, ultrasound frequency percentiles stand out as being parameters with which to discern differences between some types of honey in the temperature range studied.

### 3.2. Correlation Study

**Figure 7:**
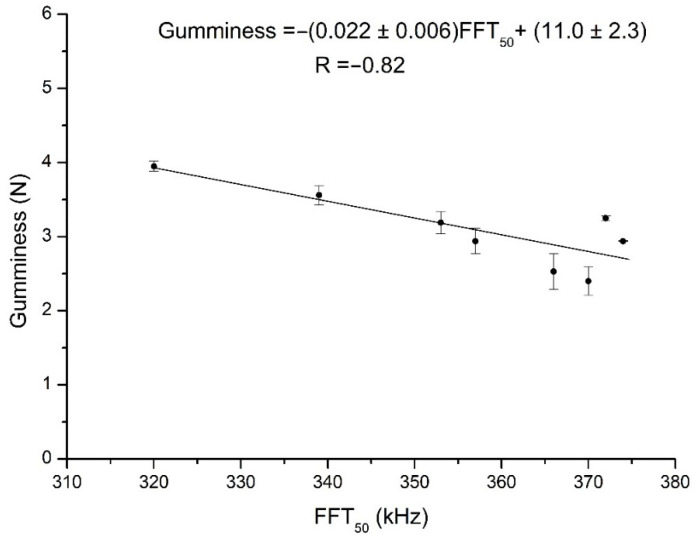
Plot of the linear regression between the gumminess and FFT_50_ values for samples measured at 30 °C.

An exhaustive analysis of the behavior of honey texture parameters has been reported in a previous work [17]. In this subsection, therefore, we shall describe the results of the linear correlation analysis between the texture parameters and the FFT parameters measured at different temperatures (Table 3). The ultrasound parameters that presented significant correlations with texture parameters were FFT_50_ and FFT_75_, although there was a dependence on temperature. FFT_50_ was negatively correlated with hardness, gumminess, and chewiness, and positively with adhesiveness at 30 °C. FFT_75_ showed the same pattern of correlations, but now at 25 °C as well as 30 °C. While FFT_25_ exhibited the same type of behavior, it was with much lower significance. The correlations for FFT_central_ were not only less significant, but even in some cases contradictory to the trends shown by the other ultrasound parameters. This once again reflects the inappropriateness of considering this parameter when characterizing honey samples, in this case in particular with regard to their texture. By way of example, Figure 7 is a plot of the linear regression analysis between gumminess and FFT_50_ at 30 °C. These results were expected because the honeys changed in consistency with temperature, being thinner at 35 °C than at 30 °C, and thinner still at 40 °C. Indeed, Alfonso et al. [20] noted that the viscosity of all kinds of honey decreases with temperature. In addition, other researchers have shown that the complex viscosity of honey samples is significantly influenced by the temperature and their soluble solids’ concentration [19,21]. In samples, such as the thicker ones, with greater amounts of undissolved solids, higher frequencies would be proportionally more attenuated than lower ones. Thus, the more fluid a honey is (and, consequently, the lower are the values of its hardness, gumminess, and chewiness, and the higher those of adhesiveness), whether because of its composition or because of its being at a higher temperature, the more easily higher ultrasound frequencies are transmitted (and hence the greater values of the FFT percentiles).

## 4. Conclusions

The frequencies of the ultrasound waves propagating through the honey samples were generally lower than the transducers’ nominal frequency. The FFT results showed a trend for higher ultrasound frequencies to be better transmitted as the honeys warm.

The statistically significant correlations found between the FFT ultrasound parameters and the texture parameters of the four honey samples add value to the utility of this non-destructive technique. Of particular interest, because of their novelty with respect to the existing literature on the topic, were the correlations observed between hardness, adhesiveness, gumminess, chewiness, and the frequency components. These correlations lend further support to the use of ultrasound for the characterization of honey since they are properties that can directly contribute to assessing the impact that a honey’s content of sugars and moisture has on its acoustic properties. Indeed, the FFT frequency components at the temperatures above 35 °C made it possible to distinguish the Thousand Flowers variety from the rest. Moreover, FFT_25_, FFT_50_, and FFT_75_ increased linearly with temperature over the range studied, and the study of the slope and intercept of these linear correlations enabled each variety to be distinguished from the others.

## Figures and Tables

**Table 1:** The mean 25th, 50th, and 75th percentile and central fast Fourier transform (FFT) frequencies (in kHz) of the received signal, and the error (eFFT). (H = Heather, Eu = Eucalyptus, Tf = Thousand Flower, T = Thyme).

Parameter	Measurement Temperature	Honey Types							
		H1	H2	Eu1	Eu2	Tf1	Tf2	T1	T2
FFT_25_	45 °C	347	373	348	341	431	468	331	357
	40 °C	330	345	326	300	391	408	313	330
	35 °C	313	333	304	270	351	356	295	291
	30 °C	300	286	288	262	287	295	273	279
	25 °C	278	273	249	259	251	258	247	249
FFT_50_	45 °C	414	430	382	408	473	537	413	423
	40 °C	401	410	359	371	440	458	395	406
	35 °C	388	393	337	352	408	413	378	381
	30 °C	372	366	320	339	353	374	357	370
	25 °C	346	340	308	317	297	304	303	313
FFT_75_	45 °C	473	483	469	467	523	641	492	479
	40 °C	461	463	436	438	491	513	450	462
	35 °C	449	446	419	418	458	477	436	441
	30 °C	431	423	415	410	416	431	419	437
	25 °C	408	406	385	381	351	363	387	392
FFT_central_	45 °C	405	434	465	404	438	477	423	440
	40 °C	351	427	365	308	433	431	400	403
	35 °C	296	416	265	226	427	427	377	409
	30 °C	357	274	270	273	276	410	268	414
	25 °C	286	270	272	278	282	275	271	272
eFFT	45 °C	4	4	3	4	6	4	3	3
	40 °C	3	6	3	4	4	5	2	3
	35 °C	3	6	3	3	4	7	2	3
	30 °C	2	4	3	3	4	7	2	3
	25 °C	3	4	4	3	4	6	4	3

**Table 2:** Linear correlations (*p* < 0.05) (slope, m; intercept, n; correlation coefficient, r) between the FFT_25_, FFT_50_, FFT_75_, and FFT_central_ and the temperature of the honeys (H = Heather, Eu = Eucalyptus, Tf = Thousand Flowers, T = Thyme).

Honey	FFT_25_			FFT_50_			FFT_75_			FFT_central_		
	m	n	r	m	n	r	m	n	r	m	n	r
H	4.3 ± 0.5	168 ± 16	0.957	3.9 ± 0.3	250 ± 10	0.980	3.54 ± 0.18	320 ± 6	0.990	7.1 ± 1.9	100 ± 70	0.801
Eu	4.4 ± 0.6	141 ± 22	0.927	4.0 ± 0.5	209 ± 17	0.948	3.9 ± 0.3	288 ± 11	0.977	7.7 ± 2.3	40 ± 80	0.765
Tf	10.0 ± 0.5	0 ± 19	0.989	9.9 ± 0.8	60 ± 30	0.973	10.6 ± 1.5	100 ± 50	0.931	10.6 ± 1.5	80 ± 70	0.857
T	4.8 ± 0.4	130 ± 13	0.976	5.1 ± 0.6	194 ± 20	0.954	4.4 ± 0.4	256 ± 15	0.965	7.6 ± 1.9	100 ± 70	0.821

**Table 3:** Correlation coefficient, r, of the linear correlations between the ultrasound FFT frequencies and the textural parameters of the honey (* *p* < 0.05).

	Temperature	Adhesiveness	Chewiness	Gumminess	Hardness
FFT_25_	35 °C	0.13	−0.05	−0.01	−0.09
	30 °C	0.11	−0.34	−0.04	−0.30
	25 °C	0.08	−0.13	−0.26	−0.19
FFT_50_	35 °C	0.28	−0.21	−0.19	−0.24
	30 °C	0.75 *	−0.81 *	−0.82 *	−0.82 *
	25 °C	0.33	−0.32	−0.51	−0.41
FFT_75_	35 °C	0.21	−0.11	−0.09	−0.17
	30 °C	0.69	−0.75 *	−0.67	−0.72 *
	25 °C	0.63	−0.58	−0.79 *	−0.69
FFT_central_	35 °C	0.63	−0.59	−0.57	−0.64
	30 °C	0.39	−0.45	−0.46	−0.42
	25 °C	−0.37	0.32	0.26	0.32

## Data Availability

Not applicable.
